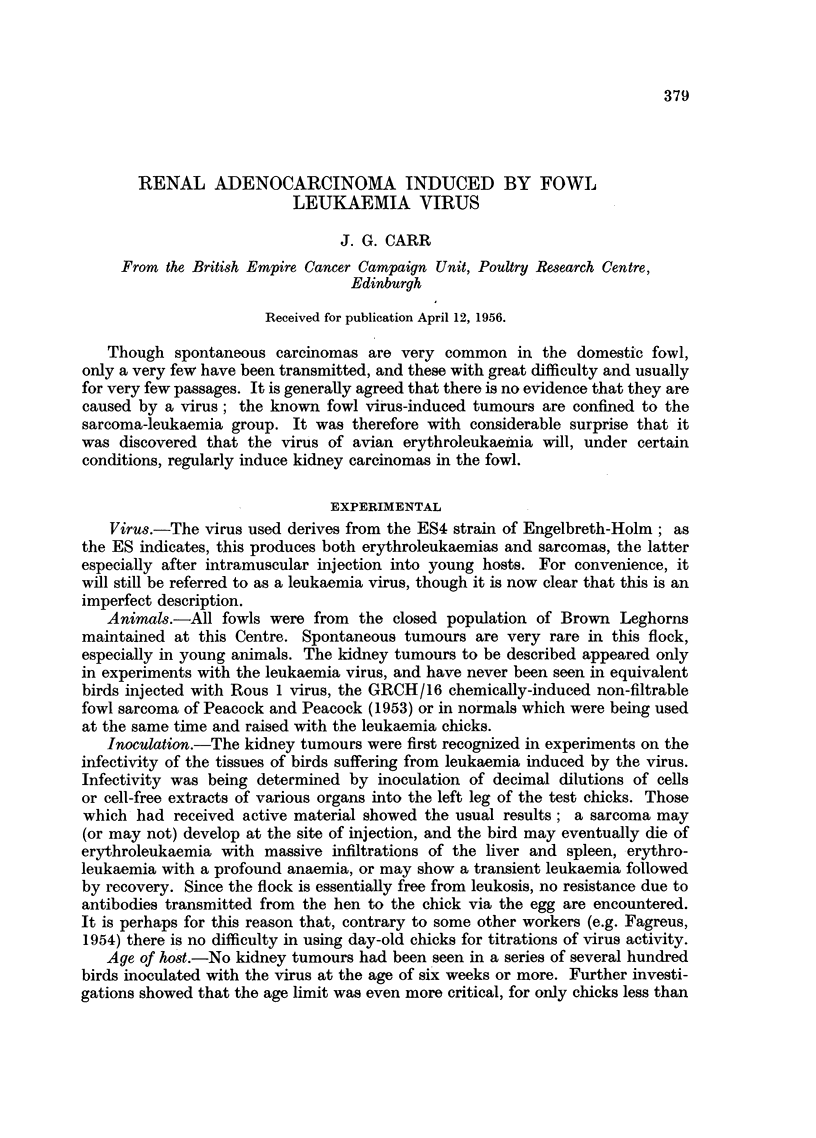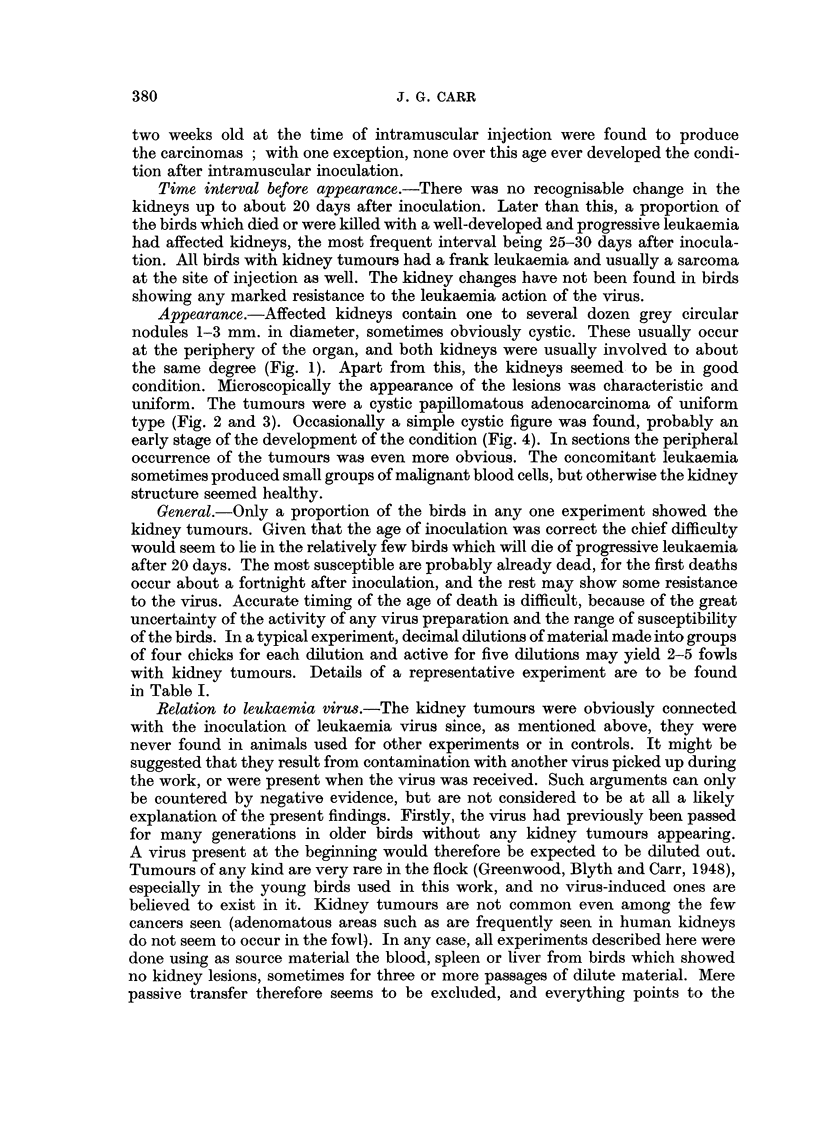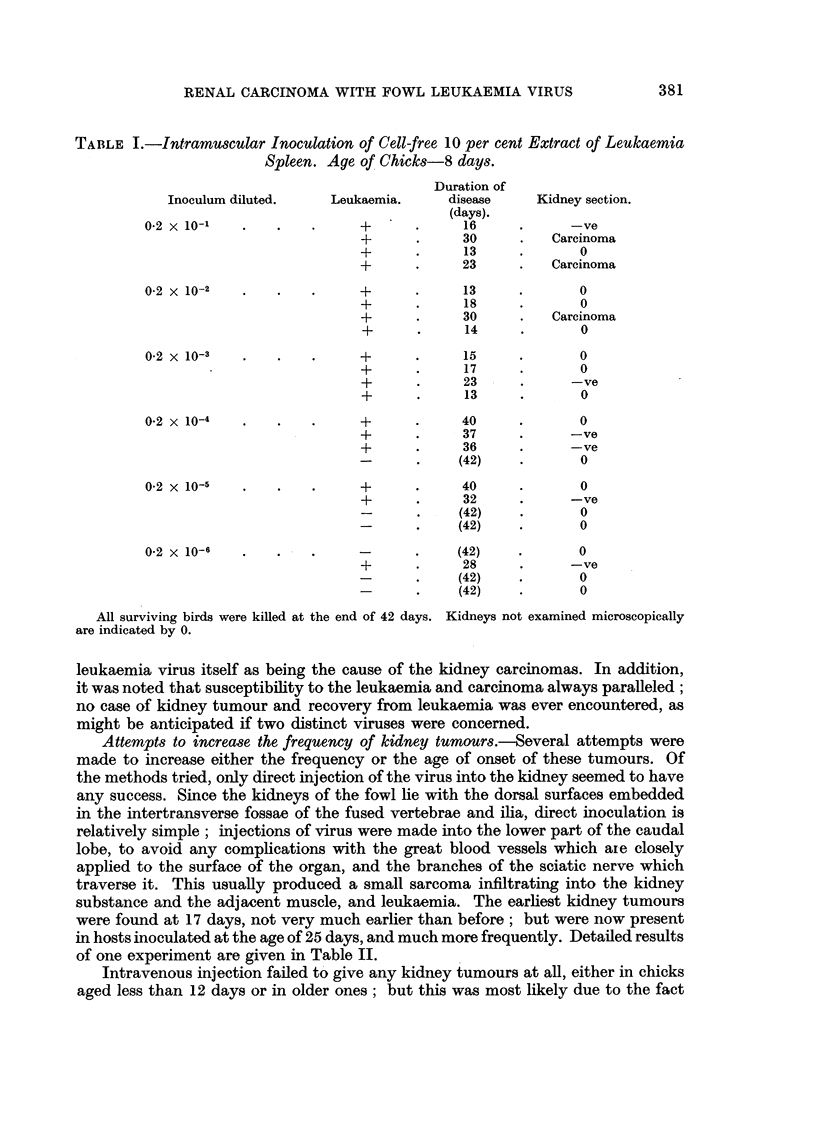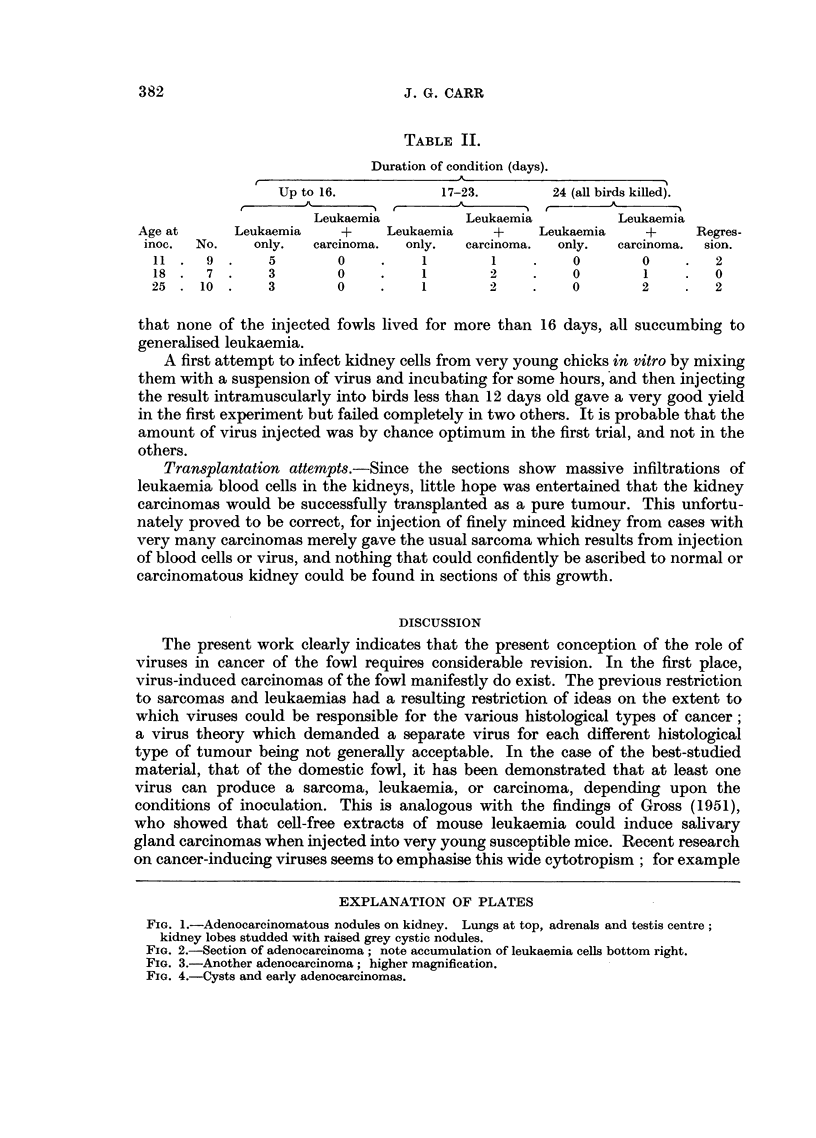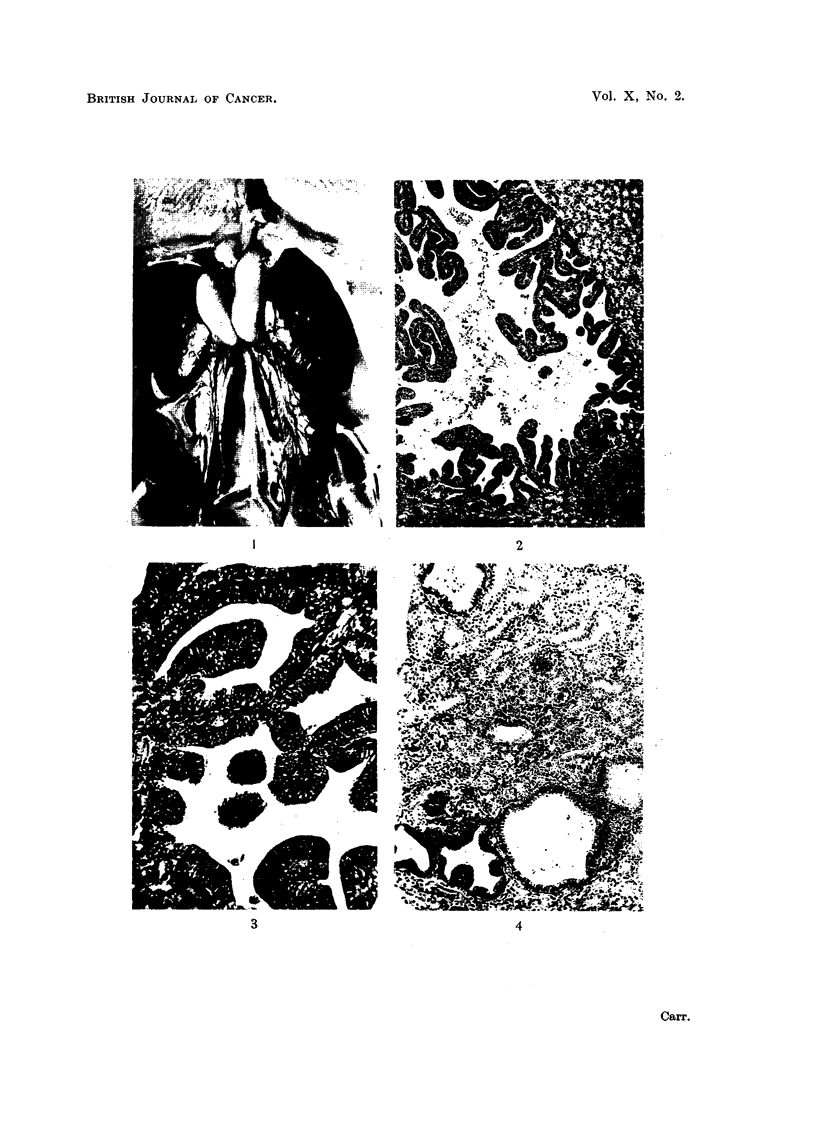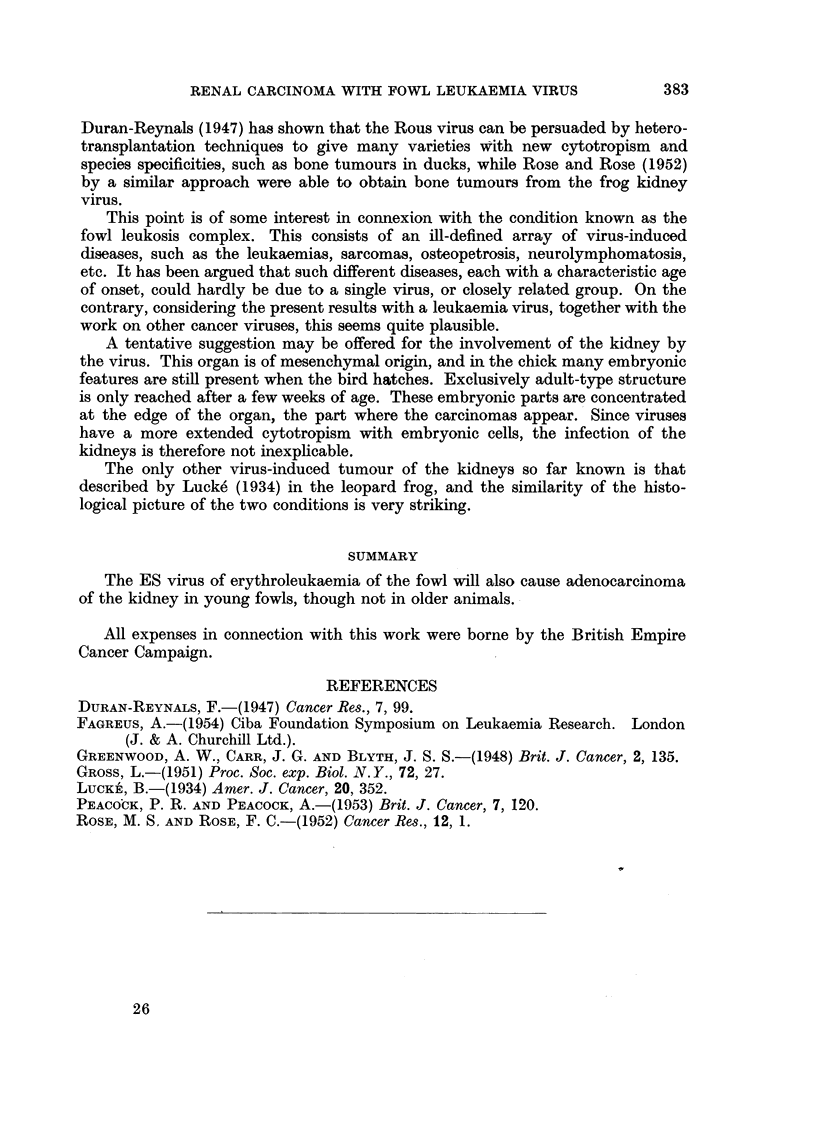# Renal Adenocarcinoma Induced by Fowl Leukaemia Virus

**DOI:** 10.1038/bjc.1956.43

**Published:** 1956-06

**Authors:** J. G. Carr

## Abstract

**Images:**


### RENAL ADENOCARCINOMA INDUCED BY FOWL LEUKAEMIA VIRUS

J. G. CARR

*From the British Empire Cancer Campaign Unit, Poultry Research Centre, Edinburgh*

Received for publication April 12, 1956.

Though spontaneous carcinomas are very common in the domestic fowl, only a very few have been transmitted, and these with great difficulty and usually for very few passages. It is generally agreed that there is no evidence that they are caused by a virus ; the known fowl virus-induced tumours are confined to the sarcoma-leukaemia group. It was therefore with considerable surprise that it was discovered that the virus of avian erythroleukaemia will, under certain conditions, regularly induce kidney carcinomas in the fowl.

### EXPERIMENTAL

*Virus.*—The virus used derives from the ES4 strain of Engelbreth-Holm ; as the ES indicates, this produces both erythroleukaemias and sarcomas, the latter especially after intramuscular injection into young hosts. For convenience, it will still be referred to as a leukaemia virus, though it is now clear that this is an imperfect description.

*Animals.*—All fowls were from the closed population of Brown Leghorns maintained at this Centre. Spontaneous tumours are very rare in this flock, especially in young animals. The kidney tumours to be described appeared only in experiments with the leukaemia virus, and have never been seen in equivalent birds injected with Rous 1 virus, the GRCH/16 chemically-induced non-filtrable fowl sarcoma of Peacock and Peacock (1953) or in normals which were being used at the same time and raised with the leukaemia chicks.

*Inoculation.*—The kidney tumours were first recognized in experiments on the infectivity of the tissues of birds suffering from leukaemia induced by the virus. Infectivity was being determined by inoculation of decimal dilutions of cells or cell-free extracts of various organs into the left leg of the test chicks. Those which had received active material showed the usual results ; a sarcoma may (or may not) develop at the site of injection, and the bird may eventually die of erythroleukaemia with massive infiltrations of the liver and spleen, erythroleukaemia with a profound anaemia, or may show a transient leukaemia followed by recovery. Since the flock is essentially free from leukosis, no resistance due to antibodies transmitted from the hen to the chick via the egg are encountered. It is perhaps for this reason that, contrary to some other workers (e.g. Fagreus, 1954) there is no difficulty in using day-old chicks for titrations of virus activity.

*Age of host.*—No kidney tumours had been seen in a series of several hundred birds inoculated with the virus at the age of six weeks or more. Further investigations showed that the age limit was even more critical, for only chicks less than two weeks old at the time of intramuscular injection were found to produce the carcinomas ; with one exception, none over this age ever developed the condition after intramuscular inoculation.

*Time interval before appearance.*—There was no recognisable change in the kidneys up to about 20 days after inoculation. Later than this, a proportion of the birds which died or were killed with a well-developed and progressive leukaemia had affected kidneys, the most frequent interval being 25–30 days after inoculation. All birds with kidney tumours had a frank leukaemia and usually a sarcoma at the site of injection as well. The kidney changes have not been found in birds showing any marked resistance to the leukaemia action of the virus.

*Appearance.*—Affected kidneys contain one to several dozen grey circular nodules 1–3 mm. in diameter, sometimes obviously cystic. These usually occur at the periphery of the organ, and both kidneys were usually involved to about the same degree (Fig. 1). Apart from this, the kidneys seemed to be in good condition. Microscopically the appearance of the lesions was characteristic and uniform. The tumours were a cystic papillomatous adenocarcinoma of uniform type (Fig. 2 and 3). Occasionally a simple cystic figure was found, probably an early stage of the development of the condition (Fig. 4). In sections the peripheral occurrence of the tumours was even more obvious. The concomitant leukaemia sometimes produced small groups of malignant blood cells, but otherwise the kidney structure seemed healthy.

*General.*—Only a proportion of the birds in any one experiment showed the kidney tumours. Given that the age of inoculation was correct the chief difficulty would seem to lie in the relatively few birds which will die of progressive leukaemia after 20 days. The most susceptible are probably already dead, for the first deaths occur about a fortnight after inoculation, and the rest may show some resistance to the virus. Accurate timing of the age of death is difficult, because of the great uncertainty of the activity of any virus preparation and the range of susceptibility of the birds. In a typical experiment, decimal dilutions of material made into groups of four chicks for each dilution and active for five dilutions may yield 2–5 fowls with kidney tumours. Details of a representative experiment are to be found in Table I.

*Relation to leukaemia virus.*—The kidney tumours were obviously connected with the inoculation of leukaemia virus since, as mentioned above, they were never found in animals used for other experiments or in controls. It might be suggested that they result from contamination with another virus picked up during the work, or were present when the virus was received. Such arguments can only be countered by negative evidence, but are not considered to be at all a likely explanation of the present findings. Firstly, the virus had previously been passed for many generations in older birds without any kidney tumours appearing. A virus present at the beginning would therefore be expected to be diluted out. Tumours of any kind are very rare in the flock (Greenwood, Blyth and Carr, 1948), especially in the young birds used in this work, and no virus-induced ones are believed to exist in it. Kidney tumours are not common even among the few cancers seen (adenomatous areas such as are frequently seen in human kidneys do not seem to occur in the fowl). In any case, all experiments described here were done using as source material the blood, spleen or liver from birds which showed no kidney lesions, sometimes for three or more passages of dilute material. Mere passive transfer therefore seems to be excluded, and everything points to the

TABLE I.—*Intramuscular Inoculation of Cell-free* 10 *per cent Extract of Leukaemia Spleen. Age of Chicks—8 days.*

| Inoculum diluted. | Leukaemia. | Duration of disease (days). | Kidney section. |
|---|---|---|---|
| $0 \cdot 2 \times 10^{-1}$ | + | 16 | —ve |
| | + | 30 | Carcinoma |
| | + | 13 | 0 |
| | + | 23 | Carcinoma |
| $0 \cdot 2 \times 10^{-2}$ | + | 13 | 0 |
| | + | 18 | 0 |
| | + | 30 | Carcinoma |
| | + | 14 | 0 |
| $0 \cdot 2 \times 10^{-3}$ | + | 15 | 0 |
| | + | 17 | 0 |
| | + | 23 | —ve |
| | + | 13 | 0 |
| $0 \cdot 2 \times 10^{-4}$ | + | 40 | 0 |
| | + | 37 | —ve |
| | + | 36 | —ve |
| | — | (42) | 0 |
| $0 \cdot 2 \times 10^{-5}$ | + | 40 | 0 |
| | + | 32 | —ve |
| | — | (42) | 0 |
| | — | (42) | 0 |
| $0 \cdot 2 \times 10^{-6}$ | — | (42) | 0 |
| | + | 28 | —ve |
| | — | (42) | 0 |
| | — | (42) | 0 |

All surviving birds were killed at the end of 42 days. Kidneys not examined microscopically are indicated by 0.

leukaemia virus itself as being the cause of the kidney carcinomas. In addition, it was noted that susceptibility to the leukaemia and carcinoma always paralleled ; no case of kidney tumour and recovery from leukaemia was ever encountered, as might be anticipated if two distinct viruses were concerned.

*Attempts to increase the frequency of kidney tumours.*—Several attempts were made to increase either the frequency or the age of onset of these tumours. Of the methods tried, only direct injection of the virus into the kidney seemed to have any success. Since the kidneys of the fowl lie with the dorsal surfaces embedded in the intertransverse fossae of the fused vertebrae and ilia, direct inoculation is relatively simple ; injections of virus were made into the lower part of the caudal lobe, to avoid any complications with the great blood vessels which are closely applied to the surface of the organ, and the branches of the sciatic nerve which traverse it. This usually produced a small sarcoma infiltrating into the kidney substance and the adjacent muscle, and leukaemia. The earliest kidney tumours were found at 17 days, not very much earlier than before ; but were now present in hosts inoculated at the age of 25 days, and much more frequently. Detailed results of one experiment are given in Table II.

Intravenous injection failed to give any kidney tumours at all, either in chicks aged less than 12 days or in older ones ; but this was most likely due to the fact

TABLE II.

| | | Duration of condition (days). | | | | | | |
|---|---|---|---|---|---|---|---|---|
| | | Up to 16. | | 17–23. | | 24 (all birds killed). | | |
| Age at inoc. | No. | Leukaemia only. | Leukaemia + carcinoma. | Leukaemia only. | Leukaemia + carcinoma. | Leukaemia only. | Leukaemia + carcinoma. | Regression. |
| 11 | 9 | 5 | 0 | 1 | 1 | 0 | 0 | 2 |
| 18 | 7 | 3 | 0 | 1 | 2 | 0 | 1 | 0 |
| 25 | 10 | 3 | 0 | 1 | 2 | 0 | 2 | 2 |

that none of the injected fowls lived for more than 16 days, all succumbing to generalised leukaemia.

A first attempt to infect kidney cells from very young chicks *in vitro* by mixing them with a suspension of virus and incubating for some hours, and then injecting the result intramuscularly into birds less than 12 days old gave a very good yield in the first experiment but failed completely in two others. It is probable that the amount of virus injected was by chance optimum in the first trial, and not in the others.

*Transplantation attempts.*—Since the sections show massive infiltrations of leukaemia blood cells in the kidneys, little hope was entertained that the kidney carcinomas would be successfully transplanted as a pure tumour. This unfortunately proved to be correct, for injection of finely minced kidney from cases with very many carcinomas merely gave the usual sarcoma which results from injection of blood cells or virus, and nothing that could confidently be ascribed to normal or carcinomatous kidney could be found in sections of this growth.

### DISCUSSION

The present work clearly indicates that the present conception of the role of viruses in cancer of the fowl requires considerable revision. In the first place, virus-induced carcinomas of the fowl manifestly do exist. The previous restriction to sarcomas and leukaemias had a resulting restriction of ideas on the extent to which viruses could be responsible for the various histological types of cancer ; a virus theory which demanded a separate virus for each different histological type of tumour being not generally acceptable. In the case of the best-studied material, that of the domestic fowl, it has been demonstrated that at least one virus can produce a sarcoma, leukaemia, or carcinoma, depending upon the conditions of inoculation. This is analogous with the findings of Gross (1951), who showed that cell-free extracts of mouse leukaemia could induce salivary gland carcinomas when injected into very young susceptible mice. Recent research on cancer-inducing viruses seems to emphasise this wide cytotropism ; for example

### EXPLANATION OF PLATES

FIG. 1.—Adenocarcinomatous nodules on kidney. Lungs at top, adrenals and testis centre ; kidney lobes studded with raised grey cystic nodules.

FIG. 2.—Section of adenocarcinoma ; note accumulation of leukaemia cells bottom right.

FIG. 3.—Another adenocarcinoma ; higher magnification.

FIG. 4.—Cysts and early adenocarcinomas.

1

2

3

4

Carr.

Duran-Reynals (1947) has shown that the Rous virus can be persuaded by heterotransplantation techniques to give many varieties with new cytotropism and species specificities, such as bone tumours in ducks, while Rose and Rose (1952) by a similar approach were able to obtain bone tumours from the frog kidney virus.

This point is of some interest in connexion with the condition known as the fowl leukosis complex. This consists of an ill-defined array of virus-induced diseases, such as the leukaemias, sarcomas, osteopetrosis, neurolymphomatosis, etc. It has been argued that such different diseases, each with a characteristic age of onset, could hardly be due to a single virus, or closely related group. On the contrary, considering the present results with a leukaemia virus, together with the work on other cancer viruses, this seems quite plausible.

A tentative suggestion may be offered for the involvement of the kidney by the virus. This organ is of mesenchymal origin, and in the chick many embryonic features are still present when the bird hatches. Exclusively adult-type structure is only reached after a few weeks of age. These embryonic parts are concentrated at the edge of the organ, the part where the carcinomas appear. Since viruses have a more extended cytotropism with embryonic cells, the infection of the kidneys is therefore not inexplicable.

The only other virus-induced tumour of the kidneys so far known is that described by Lucké (1934) in the leopard frog, and the similarity of the histological picture of the two conditions is very striking.

### SUMMARY

The ES virus of erythroleukaemia of the fowl will also cause adenocarcinoma of the kidney in young fowls, though not in older animals.

All expenses in connection with this work were borne by the British Empire Cancer Campaign.

### REFERENCES

DURAN-REYNALS, F.—(1947) *Cancer Res.*, 7, 99.

FAGREUS, A.—(1954) Ciba Foundation Symposium on Leukaemia Research. London (J. & A. Churchill Ltd.).

GREENWOOD, A. W., CARR, J. G. AND BLYTH, J. S. S.—(1948) *Brit. J. Cancer*, **2**, 135.

GROSS, L.—(1951) *Proc. Soc. exp. Biol. N.Y.*, **72**, 27.

LUCKÉ, B.—(1934) *Amer. J. Cancer*, **20**, 352.

PEACOCK, P. R. AND PEACOCK, A.—(1953) *Brit. J. Cancer*, **7**, 120.

ROSE, M. S. AND ROSE, F. C.—(1952) *Cancer Res.*, **12**, 1.